# The intestinal microbiota in enthesitis-related arthitis

**DOI:** 10.1186/1546-0096-12-S1-P47

**Published:** 2014-09-17

**Authors:** Alessia Paladini, Monica di Paola, Ilaria Pagnini, Gabriele Simonini, Teresa Giani, Paolo Lionetti, Rolando Cimaz

**Affiliations:** 1 Aou Meyer - Rheumatology Department, Florence, Italy

## Introduction

The role of gut inflammation and of intestinal permeability has been extensively studied in spondyloarthropathies, and more recently also in enthesitis--related arthritis (ERA). Clinical and experimental evidences have indicated that in genetically predisposed subjects alterations of gut microbiota may be linked to abnormal immune responses and chronic inflammatory disorders, and oral and gut microbiota have both been associated with RA.

## Objectives

In the present study, we have assessed microbial diversity in the gut of patients with ERA compared with other JIA diagnoses.

## Methods

We enrolled 27 consecutive children with JIA, 17 with ERA and 10 (as controls) with oligoarticular or polyarticular disease. The majority of cases had two separate stool samples taken at 3 months-interval. Exclusion criteria were presence of gastrointestinal symptoms and antibiotics or probiotics consumption in the month prior to sampling. Median age at sampling was 13 and 9 years, respectively, in the two groups (ranges, 8-17, and 6-14). Median disease duration was 9 y in the first group (range, 6-14) and 2 y (1-6) in the second group. In each group there were 4 pts with active disease.

From fecal samples, bacterial genomic DNA was extracted and bacterial 16S rDNA, a molecular marker used to assess microbial diversity, was amplified. All samples were subjected to Amplified Ribosomal DNA Restriction (ARDRA) analysis, a method that we had previously demonstrated to be useful in assessing microbiota dynamics on pools of genomic DNA extracted from stools from pts with with different diseases. Four restriction enzymes (HaeIII, AluI, MspI, RsaI) were selected to produce restriction fragments from amplified pools of bacterial DNA of each sample and to generate diagnostic restriction profiles. A dendrogram, based on ARDRA patterns, was constructed using the PHYLIP program with 1% tolerance (i.e. bands that differ by about seven nucleotides or less are considered identical) and with the same methodology used to compare restriction patterns from single bacterial species isolates. The clustering of the 16S rDNA restriction profiles on the mixed bacterial populations allowed discriminating every sample as unique and, in particular, to assess sample similarity and variations in the microbiota dynamics (in the same patient) according to a well-established methodology.

## Results

The dendrogram representing the clustering of different profiles obtained showed that samples from patients with ERA and those oligo- or poly- JIA clustered separately. Interestingly, HLA-B27 patients seemed to form a separate subcluster.

## Conclusion

This preliminary report shows that ERA and non-ERA patients have different stool microbiota. We are currently verifying the effects of drug treatment and disease activity on these results, and at the same time performing next generation sequencing on these samples in order to identify specific bacterial populations.

## Disclosure of interest

None declared.

